# Estimating the prevalence of 26 health-related indicators at neighbourhood level in the Netherlands using structured additive regression

**DOI:** 10.1186/s12942-017-0097-5

**Published:** 2017-07-01

**Authors:** Jan van de Kassteele, Laurens Zwakhals, Oscar Breugelmans, Caroline Ameling, Carolien van den Brink

**Affiliations:** National Institute for Public Health and the Environment - RIVM, PO Box 1, 3720BA Bilthoven, The Netherlands

**Keywords:** Small area estimation, Health-related indicators, Public health, Structured additive regression, Neighbourhoods

## Abstract

**Background:**

Local policy makers increasingly need information on health-related indicators at smaller geographic levels like districts or neighbourhoods. Although more large data sources have become available, direct estimates of the prevalence of a health-related indicator cannot be produced for neighbourhoods for which only small samples or no samples are available. Small area estimation provides a solution, but unit-level models for binary-valued outcomes that can handle both non-linear effects of the predictors and spatially correlated random effects in a unified framework are rarely encountered.

**Methods:**

We used data on 26 binary-valued health-related indicators collected on 387,195 persons in the Netherlands. We associated the health-related indicators at the individual level with a set of 12 predictors obtained from national registry data. We formulated a structured additive regression model for small area estimation. The model captured potential non-linear relations between the predictors and the outcome through additive terms in a functional form using penalized splines and included a term that accounted for spatially correlated heterogeneity between neighbourhoods. The registry data were used to predict individual outcomes which in turn are aggregated into higher geographical levels, i.e. neighbourhoods. We validated our method by comparing the estimated prevalences with observed prevalences at the individual level and by comparing the estimated prevalences with direct estimates obtained by weighting methods at municipality level.

**Results:**

We estimated the prevalence of the 26 health-related indicators for 415 municipalities, 2599 districts and 11,432 neighbourhoods in the Netherlands. We illustrate our method on overweight data and show that there are distinct geographic patterns in the overweight prevalence. Calibration plots show that the estimated prevalences agree very well with observed prevalences at the individual level. The estimated prevalences agree reasonably well with the direct estimates at the municipal level.

**Conclusions:**

Structured additive regression is a useful tool to provide small area estimates in a unified framework. We are able to produce valid nationwide small area estimates of 26 health-related indicators at neighbourhood level in the Netherlands. The results can be used for local policy makers to make appropriate health policy decisions.

## Background

From 2015 onwards, municipalities in the Netherlands were given more tasks and greater responsibilities that are of importance in the living environment of people: the so-called decentralizations of the social policy domain. As a consequence of the decentralizations, local policy makers and health care services increasingly require information on health-related indicators at smaller geographical scales, like districts or neighbourhoods.

In recent years more large data sources on health-related indicators have become available. One particular important new data source is the Dutch Public Health Monitor [1]. It is a national survey database containing figures on self-reported health, health perception, and health-related behaviours of persons aged 19 years and older. Currently, the prevalence of a large number of health-related indicators for nearly 80% of the Dutch municipalities can be provided by means of weighting methods, which are usually called ‘direct estimates’.

However, direct estimates of the prevalence of such health-related indicators cannot be produced for neighbourhoods for which only small samples or no samples are available. This implies that monitoring and target-setting can only be done at a relatively crude geographical scale. This is an important constraint for decentralized public health activities. Oversampling could provide the required information, but this is very costly. An alternative strategy to produce local estimates is to use auxiliary data, or values of the variable of interest from related areas, or both. Even when local information is not directly available, estimations for small areas can be obtained in this way. This is called small area estimation (SAE) [2]. In this paper we focus on the so-called unit-level model with a binary-valued outcome. The unit-level model uses individual observations on both the response and auxiliary data.

In recent years the statistical methodology for producing small area estimates has greatly improved. In order to capture potential non-linearities, numerically-valued predictors can be put in a SAE model in a functional form using penalized splines, although the use of P-splines in SAE models is not very common. Examples are found in [3] and [4]. A random intercept term is usually added to the model to capture heterogeneity between neighbourhoods. For area level models with a Gaussian response, the well-known Fay–Herriot model, correlated random effects can be included to account for correlation between neighbourhoods. Examples can be found in [5, 6] and [7]. However, to our knowledge, the inclusion of spatially correlated effects in the unit-level model with binary-valued outcomes is hardly ever seen, mainly because of computational issues, while it can be expected that also for binary-valued regional health-related indicators, apart from individual effects, correlation exists between adjacent neighbourhoods.

The objective of this paper is therefore twofold. First, we extend the possibilities of the Public Health Monitor and produce nationwide small area estimates of the prevalence of 26 health-related indicators at neighbourhood level in the Netherlands. Second, we present a unit-level model for binary-valued outcomes that allows us to handle both P-splines and spatially correlated random effects.

We show that it is possible to estimate the prevalence of each health-related indicator using data at the individual level. We associate the outcome with a carefully selected set of predictor variables obtained from a large national registry database. In turn, the registry data are used to predict the prevalence in the entire population. We model the associations by structured additive regression (STAR) for small area estimation, which provides a unified framework for handling both P-splines and spatial effects in the presence of non-Gaussian outcomes. See [8] for an overview, and [9] and [10] for more details. Recent computational developments make it possible to use STAR models in combination with very large datasets [11].

We validate our method by using calibration plots, in which the estimated prevalences are compared with observed prevalences at the individual level. Additionally, we compare the estimated prevalences with already available direct estimates at municipality level. Eventually, the results can be used to make appropriate health policy decisions at the local level and to respond to local care needs.

## Methods

### Municipalities, districts and neighbourhoods

In the Netherlands a municipality is an urban administrative division having corporate status and powers of self-government or jurisdiction. Municipalities are the second-level administrative division in the Netherlands and are subdivisions of their respective provinces. Their duties are delegated to them by the central government. A Municipal Health Service (MHS) is the service which every municipality must have by law in the Netherlands to carry out a number of tasks in the field of public health. Municipal Health Services work through a common system for several municipalities in a given region, called a MHS region.

For administrative use by municipalities and data collection by Statistics Netherlands (CBS), all municipalities are subdivided into districts, which in turn are subdivided into neighbourhoods. Districts and neighbourhoods have no formal status. Districts and neighbourhoods are coherent regions that are based on several characteristics like age, geographical barriers such as busy roads, having similar urban and/or architectural features, or having similar functional, social or political characteristics. As of 2012, the reference year in this paper, the Netherlands consisted of 28 MHS regions and 415 municipalities that are subdivided into 2621 districts and 11,896 neighbourhoods.

### Data sources

#### Public health monitor

The Dutch Public Health Monitor is a national survey database developed under collaboration of the National Institute for Public Health and the Environment (RIVM), the MHS’s and CBS. The database contains information on health and health perception among the Dutch population aged 19 years and older. The data were collected in 2012 on 387,195 respondents (3.0% of the Dutch population, proportionally sampled) by a questionnaire survey. It is a combination of the local monitors of the 28 MHS regions, in which 376,384 (97.2%) persons were surveyed, and the National Health Survey of the CBS, in which 10,811 (2.8%) persons were surveyed. Questions in the questionnaires and instruments were harmonized as much as possible. A secured identification number was given to each participant. It was therefore possible to link the Public Health Monitor with registry data at individual level. Authorization for this linkage has been provided by the MHS’s and CBS. Disclosure and tracing of individuals was not possible.

In this paper the following 26 health-related indicators will be considered: overweight, obesity, drinker, heavy drinker, excessive drinker, smoker, heavy smoker, diabetes, high blood pressure, asthma or COPD, joint degeneration of hips or knees, chronic arthritis, back problem, disease of the neck or shoulder, at least one chronic condition, hearing impairment, visual impairment, mobility impairment, at least one impairment, perceived good or very good health, adherence to the cardio-respiratory fitness guideline, adherence to the physical activity guideline, moderate or high risk of anxiety disorder or depression, loneliness, informal caregiver, and difficulty with making ends meet. All indicators were reported as binary-valued outcomes in the survey.

The Public Health Monitor takes place once every four years; the most recent Public Health Monitor, following the one in 2012, took place in 2016 and data will become available in 2017. More information on the Public Health Monitor, including definitions of the health-related indicators, can be found in [1].

#### Registry data

Characteristics of the Dutch population aged 19 years and older were obtained from registry data from CBS with reference date September 1, 2012. Based on expert knowledge of the MHS’s and the RIVM, 12 characteristics were chosen as possible health-related predictors. At the individual level we had age, sex, ethnicity and marital status. At household level we had household type, size, capital, income, income source and home ownership. At the neighbourhood level we had urbanization and neighbourhood code. Table 1 summarizes the population characteristics that were used as predictors in our model. Neighbourhood code was used in the model as a discrete location variable.

**Table 1:** Summary of population characteristics obtained from registry data that were used as predictors, with abbreviations that are used in the model between parentheses

Age (*age*)	Household size (*hhsize*)
Years	1, 2, …, 9, 10+
Sex (*sex*)	Household capital (*hhcap*)
Male	100 percentile classes
Female	Household income (*hhinc*)
Ethnicity (*eth*)	100 percentile classes
Autochthonous	Household income source (*hhincsrc*)
Morocco	Salaried
Turkey	Independent
Suriname	Capital
Netherlands Antilles	Unemployment benefit
Other non-western	Disability benefit
Other western	Old-age benefit
Marital status (*mar*)	Social welfare benefit
Unmarried	Other benefit
Married	Student loan
Divorced	Other
Widower	None
Household type (*hhtype*)	Home ownership (*home*)
Single person household	Homeowner
Unmarried without children	Renting with housing allowance
Married without children	Renting with no housing allowance
Unmarried with children	Neighbourhood urbanization (*urb*)
Married with children	100 percentile classes
Single parent family	Neighbourhood (*neigh*)
Other	Code

The registry data themselves had three sources. First, the municipal personal records database, which contains the characteristics of the individuals and the secured identification number, secured household identifier and secured living address identifier. Second, the household statistics database, which contains the characteristics of the households, including the secured household identifier. Third, the neighbourhood statistics database, which contains the characteristics of the neighbourhoods, including the secured living address identifier. Subsequently, all records were linked through household identifier and address identifier, resulting in one large dataset of 13,073,969 records containing characteristics at the individual level, household level and neighbourhood level.

For 345 records (0.0026%) household type and household size were missing, and for 91,669 records (0.70%) household capital, household income, household income source and home ownership were missing. These records were imputed (single imputation) by using a multinomial logit model containing age (using a natural cubic spline with knots placed on age 22, 30, 50, and 80, chosen by visual inspection), sex, ethnicity and marital status as predictors. For this purpose, the number of categories for household capital and household income was reduced from 100 to five. Given the imputed category, we then uniformly sampled one integer-valued realisation for the corresponding capital or income.

#### Direct estimates

We compared our results with already available direct estimates, which were only available at municipality level. The direct estimates were based on the Public Health Monitor database. The following weighting scheme was applied, with the number of levels between parentheses: MHS (28) × Sex (2) × Age (13) + MHS (28) × Marital status (4) + MHS (28) × Urbanization (5) + MHS (28) × Household size (5) + MHS (28) × Sex (2) × Age (3) × Marital status (2) + MHS (28) × Ethnicity (3) + MHS (28) × Income(5) + Partially merged municipality (391) × Marital status (2) + Partially merged municipality (391) × Sex (2). More information can be found in [12, 13].

### Structured additive regression model

#### Model formulation

We used a generalized structured additive regression (STAR) model to relate the predictors to the health-related indicators. Generalized STAR models provide a flexible framework for modelling (possible) nonlinear effects of the predictors on a, for example, binary-valued outcome, and allow for other effects, like spatial information. The well-established frameworks of generalized linear models and generalized additive models are considered special cases of STAR models [8].

Here, for individual *i*, *i* = 1, …, *n*, the stochastic response variable *Y*_*i*_ has a Bernoulli distribution (0/1 health-related indicator outcome) with expectation *E*(*Yi*) = *p*_*i*_, the probability of having one as outcome:

$$Y_{i} \sim Bern\left( {p_{i} } \right).$$

The relationship between *p*_*i*_ and the linear predictor *η*_*i*_ is provided by the logit link function, which is the logarithm of the odds *p*_*i*_/(1 − *p*_*i*_):

$$\log {\text{it}}\left( {p_{i} } \right) = \eta_{i} .$$

In STAR models, the linear predictor is a flexible, structured additive predictor:

$$\eta_{i} = \beta_{0} + \beta_{1} x_{i1} + \cdots + \beta_{k} x_{ik} + f_{1} \left( {z_{i1} } \right) + \cdots + f_{q} \left( {z_{iq} } \right),$$

where *x*_*i*1_, …, *x*_*ik*_ are predictors associated with individual *i* whose effect on *η*_*i*_ can be modelled through a linear predictor with unknown regression coefficients *β*_1_, …, *β*_*k*_. Typically, *x*_*i*1_, …, *x*_*ik*_ are binary-coded characteristics of an individual, such as sex, where the reference category is absorbed in the intercept *β*_0_. The functions *f*_1_(*z*_*i*1_, …, *f*_*q*_(*z*_*iq*_)) are nonlinear smooth effects of the predictors *z*_*i*1_, …, *z*_*iq*_, which are typically numerically-valued characteristics, such as age, but they also may represent spatially correlated effects. Interactions may exist between predictors, such as *β*_1_ *x*_*i*1_ + *f*_1_(*z*_*i*1_) + *f*_*z*1|*x*1_(*z*_*i*1_)*x*_*i*1_. To ensure identification of the model, it is necessary to centre the functions around zero, such that $\sum\nolimits_{i = 1}^{n} {f_{1} \left( {z_{i1} } \right) = \cdots = \sum\nolimits_{i = 1}^{n} {f_{q} \left( {z_{iq} } \right) = 0} }$ holds.

The functions *f*_*j*_(*z*_*ij*_), *j* = 1, …, *q*, are specified by a basis function approach, in which the function *f*_*j*_(*z*_*ij*_) is written as a linear combination of *d* basis functions *B*_*j*_:

$$f_{j} \left( {z_{ij} } \right) = \gamma_{1} B_{j1} \left( {z_{ij} } \right) + \cdots + \gamma_{d} B_{jd} \left( {z_{ij} } \right).$$

For numerically-valued predictors typically B-spline basis functions are chosen. B-splines are piecewise polynomials of a given degree, usually cubic, which are fused smoothly in a pre-specified number of equidistant knots. The main advantage of the B-splines basis is its local definition, i.e. being zero everywhere, except on an interval around a knot.

To prevent overfitting as the number of knots, and therefore the number of coefficients, increases, the estimation of the unknown coefficients *γ*_1_, …, *γ*_*d*_ is regularized through the introduction of a roughness penalty. These penalized B-splines are called P-splines [14]. For computational reasons, usually a quadratic penalty is assumed on the coefficients:

$$\lambda \sum\limits_{l = r + 1}^{d} {\left( {\Delta^{r} \gamma_{l} } \right)^{2} ,}$$

where *λ* ≥ 0 is an unknown smoothing parameter that controls the influence of the penalty. As *λ* → 0, the effect of the penalty disappears. ∆^*r*^ denotes *r*th order differences on the adjacent coefficients *γ*_1_, …, *γ*_*d*_. Usually *r* = 2 is chosen, as we did, which represents the discrete analogue of penalizing the second derivative of a continuous function, i.e. putting a penalty on large changes in the curvature. As *λ* → ∞, the fit approaches a polynomial of degree *r* − 1, i.e. a straight line in our case. We can write

$$\Delta ^{2} \gamma_{l} = \gamma_{l} - 2\gamma_{l - 1} + \gamma_{l - 2} ,$$

which in matrix notation can be written as a (*d* − 2) × *d* difference matrix **D**

$${\mathbf{D}} = \left( {\begin{array}{*{20}c} 1 & { - 2} & 1 & {} & {} & {} \\ {} & 1 & { - 2} & 1 & {} & {} \\ {} & {} & \ddots & \ddots & \ddots & {} \\ {} & {} & {} & 1 & { - 2} & 1 \\ \end{array} } \right),$$

where empty cells are equal to zero. This yields the penalty

$$\lambda \sum\limits_{l = r + 1}^{d} {\left( {\Delta^{2} \gamma_{l} } \right)^{2} = \lambda\varvec{\gamma}^{{\prime }} {\mathbf{D}}^{{\prime }} {\mathbf{D}}\varvec{\gamma}= \lambda\varvec{\gamma}^{{\prime }} {\mathbf{K}}\varvec{\gamma},}$$

with a *d* × *d* penalty matrix

$${\mathbf{K}} = \left( {\begin{array}{*{20}c} 1 & { - 2} & 1 & {} & {} & {} & {} \\ { - 2} & 5 & { - 4} & 1 & {} & {} & {} \\ 1 & { - 4} & 6 & { - 4} & 1 & {} & {} \\ {} & \ddots & \ddots & \ddots & \ddots & \ddots & {} \\ {} & {} & 1 & { - 4} & 6 & { - 4} & 1 \\ {} & {} & {} & 1 & { - 4} & 5 & { - 2} \\ {} & {} & {} & {} & 1 & { - 2} & 1 \\ \end{array} } \right).$$

A similar principle of penalized basis functions applies to spatial data as well. For regional health-related indicators, it can be expected that, apart from individual and household effects, spatial heterogeneity may exist. In our case we had discrete spatial information in the form of neighbourhoods, where correlation may exist between adjacent neighbourhoods.

For data observed on a regular or irregular lattice, a common approach for the correlated spatial effect is based on Markov random fields [15]. Each individual *i* belongs to a particular neighbourhood *s*. A regression coefficient *γ*_*s*_ is assigned to each neighbourhood *s*, *s* = 1, …, *d*. The corresponding basis function *B*_*is*_ is 1 if individual *i* belongs to neighbourhood *s*, and is 0 otherwise. Adjacent neighbourhoods are usually defined by common boundaries (Rook type contiguity). We use the notation *s* ~ *r* to denote that neighbourhoods *s* and *r* are adjacent. The penalty again consists of squared differences and can compactly be written as *λ* ***γ***′**K** ***γ***, where **K** is a *d* × *d* matrix with elements **K**_*sr*_ = −1 if *s* ≠ *r*, *s* ~ *r*, **K**_*sr*_ = 0 if *s* ≠ *r*, *s* ≁ *r*, and **K**_*sr*_ = |*N*(*s*)| if *s* = *r*, and where |*N*(*s*)| is the number of adjacent neighbourhoods of *s*. In other words, large first order differences between adjacent neighbourhoods are penalised. For example, for *d* = 9 neighbourhoods in a regular 3 × 3 lattice, numbered from left to right and from top to bottom, the penalty matrix **K** is given by

$${\mathbf{K}} = \left( {\begin{array}{*{20}c} 2 & { - 1} & {} & { - 1} & {} & {} & {} & {} & {} \\ { - 1} & 3 & { - 1} & {} & { - 1} & {} & {} & {} & {} \\ {} & { - 1} & 2 & {} & {} & { - 1} & {} & {} & {} \\ { - 1} & {} & {} & 3 & { - 1} & {} & { - 1} & {} & {} \\ {} & { - 1} & {} & { - 1} & 4 & { - 1} & {} & { - 1} & {} \\ {} & {} & { - 1} & {} & { - 1} & 3 & {} & {} & { - 1} \\ {} & {} & {} & { - 1} & {} & {} & 2 & { - 1} & {} \\ {} & {} & {} & {} & { - 1} & {} & { - 1} & 3 & { - 1} \\ {} & {} & {} & {} & {} & { - 1} & {} & { - 1} & 2 \\ \end{array} } \right).$$

The linear predictor can now be written as follows:

$$\begin{aligned} \eta_{i} = & \beta_{0} + \beta_{sex}\,sex_{i} + f_{age} \left( {age_{i} } \right) + f_{age|sex} \left( {age_{i} } \right) + \mathop \sum \limits_{j = 1}^{6} \beta_{eth,j}\,eth_{ij} \\ & + \mathop \sum \limits_{j = 1}^{3} \beta_{mar,j}\,mar_{ij} + \mathop \sum \limits_{j = 1}^{6} \beta_{hhtype,j}\,hhtype_{ij} + f_{hhsize} \left( {hhsize_{i} } \right) + f_{hhcap} \left( {hhcap_{i} } \right) \\ & + f_{hhinc} \left( {hhinc_{i} } \right) + \mathop \sum \limits_{j = 1}^{10} \beta_{hhincsrc,j}\,hhincscr_{ij} + \mathop \sum \limits_{j = 1}^{2} \beta_{home,j}\,home_{ij} \\ & + f_{urb} \left( {urb_{i} } \right) + f_{neigh} \left( {neigh_{i} } \right) \\ \end{aligned}$$

The non-linear functions of all numerically-valued predictors were modelled by cubic B-splines basis functions with 10 knots and a penalty on the second order differences of the coefficients, except for household size, which had five knots. These numbers were based on preliminary analyses. The choice of the number of knots was not critical, but it was important not to make it restrictively small, nor very large and computationally costly. Furthermore, the spatial correlation between neighbourhoods was taken into account. Although the number of regression coefficients was large, the effective number of coefficients was usually much lower because of the smoothing penalties. The amount of smoothing was selected automatically as will be explained in the next section.

#### Parameter estimation

All data preparations and analyses were carried out in R [16], using the data.table package for handling the large datasets [17] and the sp and maptools packages for handling the spatial data [18, 19]. Estimation of parameters was carried out via restricted maximum likelihood (REML) in the R package mgcv [10, 20].

Because of the large dataset in combination with the model’s complexity, it was impossible to fit the model to the whole dataset. Therefore the dataset was split by MHS region, and for each combination of MHS region (28) and health-related indicator (26) a model was run. We combined MHS regions *GGD Drenthe* and *GGD Groningen*, located in the north-east of the Netherlands, because the number of respondents in *GGD Drenthe* appeared too low for a proper estimation of the regression coefficients for those regions separately. So, in total there were 27 × 26 = 702 model runs. For each run, the same model formulation was used. The fitted models differed only in their sets of estimated regression coefficients and smoothing parameters.

To avoid boundary effects, first a 10 km buffer was created around each MHS region using the rgeos package [21]. Neighbourhoods (and all individuals within) with their centroid located within the buffer were included in the estimation procedure. Next, a neighbourhood adjacency list (graph) was created, based on neighbourhoods with contiguous boundaries, using the spdep package [22]. The creation of the 10 km buffer sometimes resulted in artificial islands that were disconnected with the considered region, i.e. subgraphs. Besides, natural islands are also a common feature in the Netherlands. To avoid a disconnected graph, neighbourhoods in two unconnected subgraphs that were located the closest to each other were connected, using the RANN package [23]. The Euclidian distance between centroids was taken as distance measure. This was repeated until there was only one connected graph left.

**Fig. 1:**
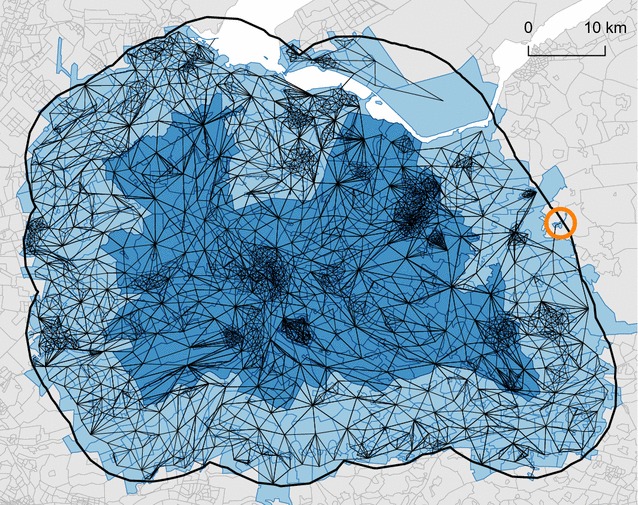
Illustration of the construction of the 10 km buffer (*light blue area*) and adjacency list (*thin black lines*) for the MHS regions ‘GGD Midden Nederland’ and ‘GG en GD Utrecht’ (*dark blue area*). An artificial island is marked by the *orange circle*. North is up

The construction of the 10 km buffer and the adjacency list is illustrated in Fig. 1. The region corresponds to the Dutch province of Utrecht (for illustrative purpose the MHS regions ‘*GGD Midden Nederland’* and ‘*GG en GD Utrecht’* were combined here) and is indicated by the dark blue colour. The thick black line indicates the 10 km buffer and the additional neighbourhoods that were included in the estimation procedure are indicated by a light blue colour. Adjacent neighbourhoods are connected by a black line. In the east, indicated by an orange circle, an artificial island can be seen, now connected with the rest of the region.

Although the splitting of the dataset by MHS region reduced the number of records considerably, the introduction of the 10 km buffer and inclusion of the spatial and heterogeneity terms in the model still resulted in very large numbers to handle. The following numbers are averages for each model run: 34,782 individuals and 975 neighbourhoods were used for estimation, 1052 regression coefficients were estimated and 484,221 individuals and 441 neighbourhoods were predicted. For this reason, we used the bam function in mgcv, which is much like the standard gam function in mgcv, except that the numerical methods are designed for very large datasets. The advantage of bam is a much lower memory footprint than gam, but it can also be much faster and can be run in parallel [11].

#### Prediction

Once the regression coefficients for a combination of a MHS region and health-related indicator were estimated, the formula for the linear predictor was applied to all individuals in the registry data for that region. This resulted in a log-odds of having one as an outcome for each individual. The log-odds were subsequently transformed into probabilities. Because on average the outcome was actually known for 3.0% of the individuals, the probabilities for these individuals were replaced by their observed binary-valued outcome, as is the common procedure in small area estimation [2]. The individual outcomes were aggregated to neighbourhood (11,432), district (2599) and municipality level (415) to obtain prevalence estimates. To prevent disclosure or privacy issues, results for regions with fewer than 10 inhabitants aged 19+ were sanitised (i.e. not reported). This was the case for 464 neighbourhoods and 22 districts.

Each estimation and prediction step took on average 3 m 19 s to run on an Intel^®^ Xeon^®^ X5560 2.80 GHz CPU with four sockets running a 64-bit Windows 7 operating system. One health-related indicator took 1 h 30 m to run. The whole exercise took 1 day 14 h 51 m to run.

### Validation

#### Calibration plots

The model’s validity was checked using calibration plots. Calibration refers to the agreement between estimated outcomes and observations. In a calibration plot the estimated prevalences is compared with the observed prevalence [24]. For example, if the model predicts that a respondent has a 20% probability of having overweight, the observed frequency of overweight should be approximately 20 out of 100 respondents with such a prediction.

Here, the 387,195 respondents were randomly split in 2/3 training individuals and 1/3 validation individuals. Next, for each health-related indicator, the model was fitted to the training dataset as described in the previous section (i.e. same model formulation and estimation procedure, and stratification by MHS region). Subsequently, the estimated regression coefficients were used to make predictions for all individuals in the validation dataset. Because it is impossible to compare a predicted probability with a binary response at the individual level, the predicted probabilities in the validation dataset were divided into 200 equally sized intervals according to their quantiles. Then for each interval, the predicted probabilities and corresponding observed binary responses were averaged, resulting in an averaged predicted prevalence and an observed prevalence for that interval, which can be compared in a scatterplot [24]. The points in the scatter-plot should lie near the 1:1 line. It is assumed that if the model can accurately predict the indicator of an individual, then at neighbourhood, district or municipality level the estimated prevalence will be close to the true prevalence.

#### Comparison between small area estimates and direct estimates

At municipality level we compared the small area estimates with the already available direct estimates, obtained by weighted estimation. Since the true prevalence is unknown, this is not an actual validation, but it can give an impression of how well the small area estimates agree with the direct estimates.

## Results

We illustrate the small area estimation procedure for overweight. First we consider the province of Utrecht, represented in Fig. 1, for which we show the estimated regression coefficients. Other regions are alike. Next, we show the overweight prevalence for all 11,896 neighbourhoods in the Netherlands. We end with assessing the model’s performance.

### Estimated effect sizes

**Fig. 2:**
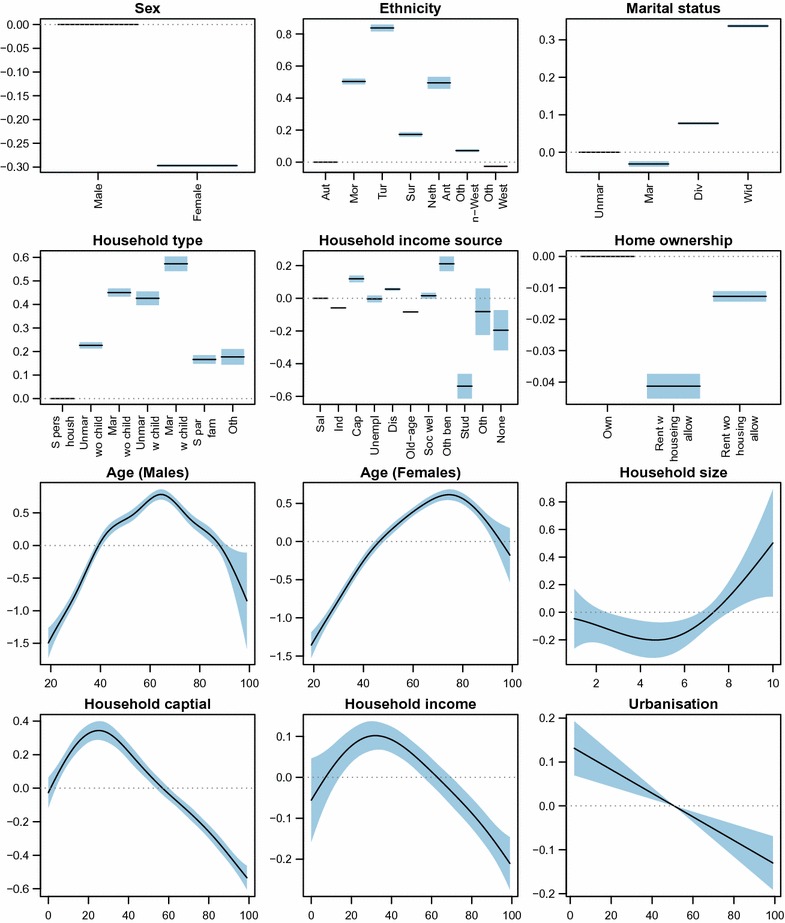
Estimated regression coefficients for the overweight model for the province of Utrecht (MHS regions ‘GGD Midden Nederland’ and ‘GG en GD Utrecht’). Levels or values of each predictor are given on the *x*-axis. The estimated effect size, in terms of differences in log-odds compared to the reference category or value, is given on the *y*-axis. The *shaded areas* represent the 95% confidence intervals. The reference category is always on the *horizontal dotted line* at zero

Figure 2 shows the estimated regression coefficients corresponding to the categorical predictors (top six panels) and the smooth terms corresponding to the numerically-valued predictors (bottom six panel), corresponding to the terms in the formula for the linear predictor. The values can be interpreted as differences in log-odds. Similar patterns for overweight are visible in other MHS regions (figures not shown) and similar graphs can be made for the other health-related indicators (figures not shown). Note that it is not the goal of this paper to explain effect sizes and differences. Here, they are solely used to make the predictions.

Compared to males, females have lower overweight prevalence, but there is also a strong interaction with age. Compared to the autochthonous population, most ethnicities have higher prevalence, especially people with a Turkish background. Compared to (un)married people, divorced or widowed people have higher prevalence. Compared to single-person households, other household types have a higher prevalence. Compared to households having a salaried income, households having their income from capital and other benefits have higher prevalence. On the other hand, student households have lower prevalence. Home ownership is not an important predictor for overweight, as can be seen from the small differences between the three categories. Age is an important predictor for overweight. Younger people have a lower prevalence, whereas the prevalence increases with age, reaching a maximum around 65 years for men and 75 years for women. For the elderly, especially men, the prevalence decreases again. The prevalence increases with household size. Households with a lower capital or income have higher prevalence, whereas the prevalence decreases with increasing capital and income. Finally, the prevalence decreases with increasing urbanisation (note: although the effect of urbanisation is modelled with a P-spline, the relation is estimated to be a straight line. The disappearing of the 95% confidence interval near the mean is a result of the sum-to-zero identifiability constraints).

**Fig. 3:**
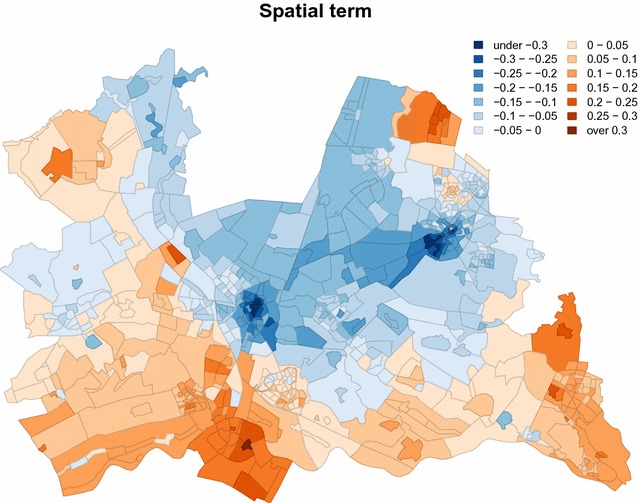
Estimated spatial term for the overweight model for the province of Utrecht (MHS regions ‘GGD Midden Nederland’ and ‘GG en GD Utrecht’). *Blue colours* indicate lower log-odds compared to the expected log-odds, *orange colours* higher log-odds

Figure 3 shows for the province of Utrecht the estimated spatial effect, corresponding to the last term in the formula for the linear predictor. The term represents the estimated spatial heterogeneity, apart from individual, household and urbanisation effects. Blue colours indicate lower overweight prevalence than expected. Orange colours indicate higher overweight prevalence than expected. Clear geographic patterns are visible. In the large cities Utrecht and Amersfoort, respectively located in the centre and in the north east, the prevalence is lower than expected. In the rural areas in the south, the prevalence is higher than expected. Note that it is not the goal of this paper to explain these patterns.

### Prevalence map

**Fig. 4:**
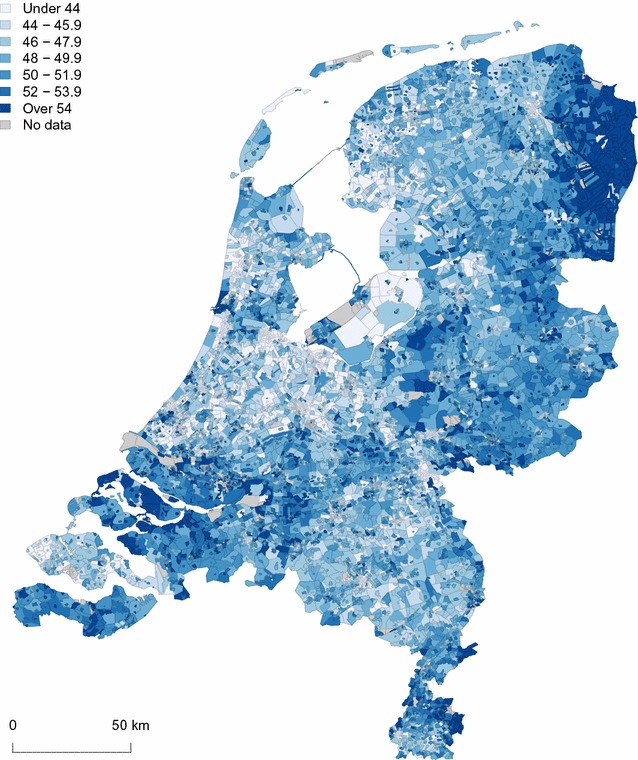
Map of the estimated overweight prevalence in percentages at neighbourhood level in The Netherlands. Neighbourhoods with less than 10 inhabitants are sanitised (*grey*). North is up

Figure 4 shows a map of the estimated overweight prevalence (%) at neighbourhood level in the Netherlands. The darker the colour, the higher the prevalence. Neighbourhoods with fewer than 10 inhabitants aged 19+ are indicated with “No data”. Although differences are small, there are distinct geographic patterns visible. High prevalences are especially seen in the north-eastern and south-western part of the Netherlands. Other clusters of high prevalence are seen at the ‘Veluwe’, a forested area just east of the country’s centre, and in the south-east, around the so-called ‘Parkstad’, a former mining colony. Lower overweight prevalences are found elsewhere.

### Assessing the model’s performance

**Fig. 5:**
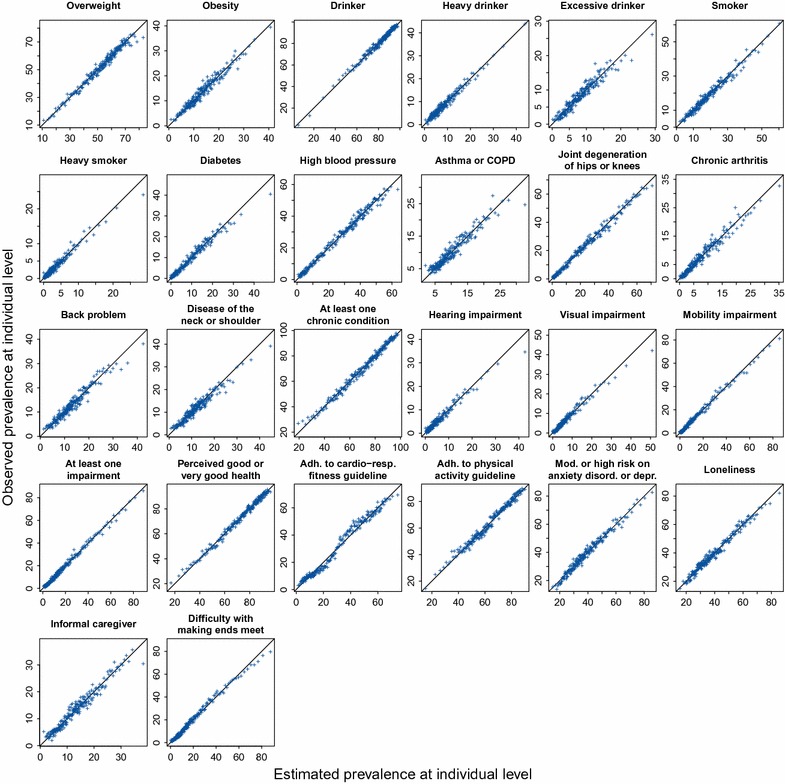
Calibration plots of the 26 health-related indicators. Estimated prevalence is given on the *x*-axis, observed prevalence on the *y*-axis. Each *dot* is the average of 645 individuals

Figure 5 shows calibration plots to see how well the model is able to predict the prevalence of the 26 health-related indicators at the individual level. Each dot represents the average of about 645 respondents. On the x-axis the predicted prevalence is shown, on the y-axis the corresponding observed prevalence. The diagonal line is the 1:1 line. All dots are close to the 1:1 line over the whole prevalence range for almost all health-related indicators. This indicates that the model is very capable of predicting the prevalence at the individual level.

**Fig. 6:**
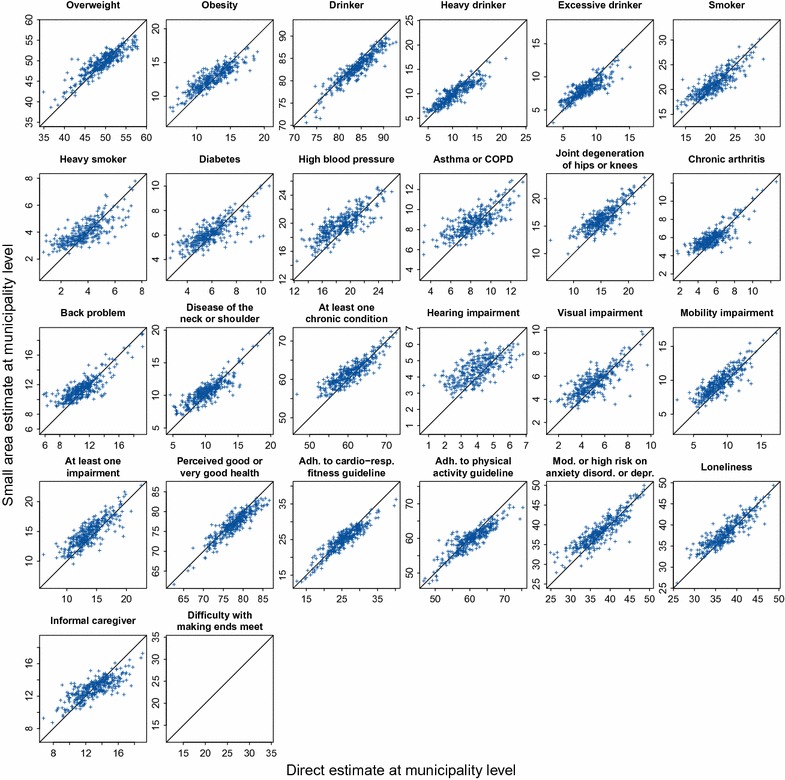
Small area estimates on the *y*-axis compared to the direct estimates on the *x*-axis. Each *dot* is a municipality. For one health-related indicator no direct estimates were available

Figure 6 shows the small area estimates on the y-axis compared to the direct estimates on the x-axis. Each dot represents a municipality. For the health-related indicator ‘Making ends meet’ no direct estimates were available. There exists a moderate correlation between the two estimates. There seems to be a tendency for the small area estimates being both somewhat higher and less extreme than the direct estimates.

## Discussion

### STAR models in small area estimation

We have shown that it is possible to extrapolate the data from the Public Health Monitor by providing estimates of the prevalence of 26 health-related indicators for 11,896 neighbourhoods, 2621 districts and 415 municipalities in the Netherlands. This was done by relating each indicator to a given set of predictors at the individual, household and neighbourhood level, obtained from registry data. We have used a generalized structured additive regression model, which is, to our knowledge, a relatively new concept in SAE. Another application of STAR models in SAE can be found in [25].

STAR models allow modelling of non-linear relationships using P-splines and modelling spatially correlated data in a unified way. This is accomplished by using basis functions, of which the corresponding regression coefficients are automatically regularized through a roughness penalty to prevent overfitting. We have seen that, for the numerically-valued predictors, effects can be non-linear. There was usually enough information in the data to pick up such signals, as on average 34,782 individuals and 975 neighbourhoods were used for estimation. If for some reason no relationship exists, the roughness penalty becomes so high that the result is a (non-significant) straight line, as we have seen for the relation between household size and overweight.

We have seen that the individuals’ characteristics alone are not able to explain the outcome entirely. Still (spatial) patterns exist that are unaccounted for, which should be included as additional random effects in the model. This is often encountered in hierarchical Bayes SAE models (e.g. [2]). The STAR modelling approach treats these random effects in a similar way as the numerically-valued predictors. The amount of smoothing is automatically determined by the amount of information in the data. A spatially correlated random effect has the additional advantage that it interpolates to neighbourhoods where no individuals are sampled. It borrows information from adjacent neighbourhoods in a similar way the P-splines do for numerically-valued predictors. However there still may exist additional heterogeneity between neighbourhoods that cannot be described by individual or spatial effects. This effect is usually modelled by a random intercept, and in the STAR framework it can be similarly modelled as a spatial random effect, except that the penalty matrix **K** simplifies to a *d* × *d* identity matrix, which is the classical ridge penalty [8]. We investigated whether a random intercept should be included in the model, but no effect was found.

The creation of a 10 km buffer around each MHS region seemed necessary. Without the buffer, unrealistic clear-cut boundaries between MHS regions appeared in the results. Although the buffer considerably diminished boundary effects, still some regional effects could be recognized because of the stratification by MHS region, even though the data collection and cleaning were harmonized between MHS regions.

### Accuracy of the model

The accuracy of the model’s estimate has been checked by calibration plots and by comparing the small area estimates with the direct estimates. The binary-valued survey data at the individual level are the best we have to compare the estimates with. The model tries to follow the survey data as well as possible. The calibration plots showed that the model is very capable of estimating the prevalence of the 26 health-related indicators at the individual level. Although not guaranteed, since the true prevalence at neighbourhood level cannot be observed, this suggests that the prevalence at neighbourhood level (or at district or municipality level) may be very close to the true prevalence.

The small area estimates agree reasonably well with the direct estimates already available at municipality level. Although the set of predictors that were accounted for in the weighting scheme showed many similarities with our selected set, we should realise that two totally different estimation procedures are compared here. We additionally included household type, household capital, household income source, and home ownership as predictors in our model and the numerically-valued predictors have not been categorized into an arbitrary number of classes.

### Plausibility of the estimates

Plausibility of the estimates was checked in two ways: first, by identifying and explaining extreme estimates, and second, by asking feedback from the Municipal Health Services.

For each health-related indicator we identified the most extreme estimates, i.e. identified the healthiest and unhealthiest neighbourhoods. We located the neighbourhoods on a map and investigated the properties of these neighbourhoods and the residents within. First we noticed that the extremes are not caused by a low number of residents in the neighbourhood; almost all identified outlying neighbourhoods consisted of around 30–300 residents. Instead, extremes were caused by homogeneous groups of individuals sharing the same characteristics that are associated with high or low prevalences, for example, elderly, or people of a certain ethnicity. There was often some institution present in the neighbourhood, e.g. a nursing home, an institute for the mentally disabled, a university campus or an asylum centre. Note that the associations are based on all sampled individuals in a MHS region. A neighbourhood with a homogeneous group of typical individuals then subsequently results in an extreme prevalence. We think it is a good sign that the model is able to pick up such signals.

The feedback from the MHS’s, whether the estimates make sense based on their familiarity with the local situation, was mostly positive. Note that this was a qualitative assessment. The estimates did agree with what was expected, although it was noticed, that the small area estimates were less extreme than the direct estimates (when available). The expected differences between neighbourhoods were noticeable and logical. Deprived neighbourhoods, often associated with an unhealthy lifestyle, were in the top rank as expected.

Some results, however, were remarkable. For example, for one neighbourhood that mainly consisted of students the model estimated the prevalence of smokers to be 44%, heavy smokers 12%, and risk of anxiety disorder or depression 49%. This was doubted by the MHS. After further investigation we found that most residents were 19–25 years old, were of an ‘other Western’ background, were mostly unmarried, and living in single person households. The household capital and income was mainly (very) low, and if any income was generated, it was by employment or a student benefit. Home ownership was mainly rent without housing allowance. By looking at the associations between smoking behaviour and the predictors (as described by Fig. 2), we saw that smoking was associated with young age, small households, low household capital and income, and rent without housing allowance. These associations are based on all participants in the corresponding MHS. It is therefore not surprising that the model subsequently produced such a high smoking prevalence for this neighbourhood.

What is missing is educational level, which is usually considered as a strong predictor for health-related indicators. Household capital, income, income source and home ownership were used as a proxy, but for this specific neighbourhood this seemed to be insufficient. Furthermore, the estimate is the product of an expected value based on the individual’s characteristics and a spatial random effect at neighbourhood level. The latter can be estimated better if more people would have participated in the Public Health Monitor in that neighbourhood (currently only two). With less information, the estimate is shrunken towards the expected value based on individual’s characteristics and the random effect borrows more information from of the surrounding neighbourhoods. In other words, if the information was there, then we would have gotten a better estimate for this specific neighbourhood.

### Limitations and possible extensions

This brings us to the limitations and possible extensions of the model. First, sufficient data should be available; registry data as well as survey data. In the Netherlands, high-resolution registry data is available (in a secured environment). Such data may not be available in other countries. We have also noticed that educational level should have been included as a predictor. However this predictor is only partly available in the registry data and therefore cannot be included. This may improve in the future as more data on educational level becomes available. Next to appropriate registry data, still sufficient survey data are needed to be able to associate the health-related indicators with the predictors. Here, conveniently, 387,195 participants were incorporated that were proportionally sampled throughout the Netherlands. We have not investigated what the minimum number of respondents would be in relation to the number of predictors and neighbourhoods, but this may be a topic for further research.

The availability of appropriate registry data is related to the selection of predictors. Here selection was done by expert knowledge. We have not considered applying any variable selection methods, e.g. [26], but this may be also a topic for further research. As there is no information on the non-responders, we had to assume that the predictors were evenly distributed across responder and non-responders. However, we realize that survey research frequently involves some selection bias.

One must bear in mind that inappropriate modelling choices may produce incorrect results. For instance, our methodology could be applied to other indicators, e.g. experienced noise annoyance, which could be associated with local environmental exposure to noise. However, if local exposure to noise is not included as a predictor in the model and an association is found with e.g. low household capital, this does not automatically mean that all individuals with low household capital experience noise annoyance.

Although recent computational developments make it possible to use STAR models in combination with very large datasets [11], it is currently unfeasible to provide estimates of prediction uncertainties. The model does provide uncertainty estimates at the individual level, but aggregation to neighbourhood, district or municipality level causes difficulties. In theory Monte Carlo simulation could be a solution: (1) Draw a large number, say 1000, realisations of the estimated regression coefficients from a multivariate Normal distribution, which takes care of the covariances. (2) For a given set of coefficients, predict the prevalences at the individual level. (3) For each given prevalence, draw a realisation of the outcome from a Bernoulli distribution. Replace the realisation by the observed response for individuals whose outcome is actually known. (4) For each neighbourhood, add up the individuals’ outcome and divide by the total number of individuals in that neighbourhood. (5) Calculate the desired summary statistics, e.g. mean or standard deviation. The main problem is that these steps have to be carried out for 13,073,969 individuals. In combination with, on average, 1052 regression coefficients, this results in a huge linear predictor matrix. This is computationally infeasible at the moment and may be a topic for further research.

## Conclusions

The possibilities of Public Health Monitor can be extended to produce nationwide small area estimates of 26 health-related indicators at neighbourhood level in the Netherlands. Registry data is needed to make predictions. Structured additive regression is a useful tool to provide small area estimates in a unified framework. The model can handle both non-linear relations and spatial effects in the presence of binary-valued outcomes.

The model tries to follow the survey data as well as possible. The estimated prevalences agree very well with observed prevalences at the individual level. The estimated prevalences agree reasonably well with the direct estimates at the municipal level. The estimates seem plausible. The results can be used by local and policy makers to make appropriate health policy decisions at the local level and by health care services to respond to local care needs.

## Abbreviations

CBS: Centraal Bureau voor de Statistiek (Statistics Netherlands)
COPD: chronic obstructive pulmonary disease
GGD: Gemeentelijke Gezondheidsdienst (Municipal Health Service)
MHS: Municipal Health Service
REML: restricted maximum likelihood
RIVM: Rijksinstituut voor de Volksgezondheid en Milieu (National Institute for Public Health and the Environment)
SAE: small area estimation
STAR: structured additive regression
